# Supplementary material for: Predictors of Psychological Distress across Three Time Periods during the COVID-19 Pandemic in Poland
Source: Int J Environ Res Public Health. 2022 Nov 21;19(22):15405. doi: 10.3390/ijerph192215405 (PMC9690834; doi:10.3390/ijerph192215405)
Supplement: Supplementary file 1 [file ijerph-19-15405-s001.zip › ijerph-1980110-supplementary.pdf]

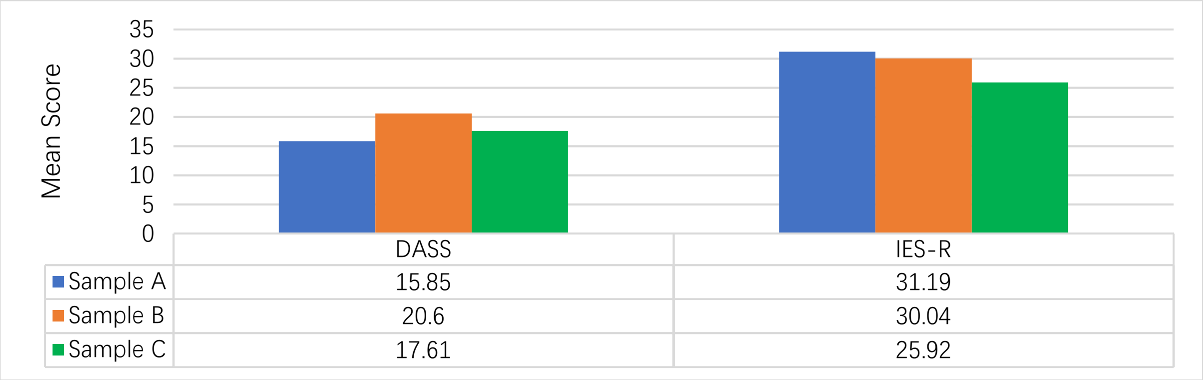

Figure S1. Comparison of the mean scores of DASS-stress, anxiety and depression subscales and the IES-R scores between three surveys.

Table S1. Comparison of physical symptoms related to COVID-19 and health status between the first, second and third-survey respondents (n =2324).

Physical symptoms and health status	The first-survey respondents (N = 1064)	The second-survey respondents (N = 557)	The third- survey respondents (N = 703)	Chi-square statistics (X^2)	p-value
	N (%)	*N (%)*	*N (%)*		
**Fever**					
Yes	40 (3.8)	50 (9)	40 (5.7)	18.862	<0.001***
**Cough**					
Yes	165 (15.5)	143 (25.7)	128 (18.2)	24.992	<0.001***
**Chills**					
Yes	32 (3)	39 (7)	43 (6.1)	15.675	<0.001***
**Myalgia**					
Yes	72 (6.8)	91 (16.3)	118 (16.8)	52.398	<0.001***
**Breathing Difficulty**					
Yes	36 (3.4)	55 (9.9)	39 (5.5)	29.172	< 0.001***
**Coryza**					
Yes	266 (25)	163 (29.3)	166 (23.6)	5.584	0.061
**Sore throat**					
Yes	204 (19.2)	105 (18.9)	129 (18.3)	0.187	0.911
**Fatigue**					
Yes	301 (28.3)	254 (45.6)	366 (52.1)	110.916	< 0.001***
**No complains**					
Yes	506 (47.6)	222 (39.9)	264 (37.6)	19.709	< 0.001***
**Consultation with a doctor in the clinic in the past 14 days**					
Yes	245 (23)	153 (27.5)	207 (29.4)	9.842	0.007**
**Recent hospitalization in the past 14 days**					
Yes	18 (1.7)	16 (2.9)	10 (1.4)	3.947	0.139
**Recent quarantine in the past 14 days**					
Yes	6 (0.6)	42 (7.5)	21 (3)	61.769	<0.001***

**Recent testing for COVID-19 in the past 14 days**					
Yes	244 (23)	46 (8.3)	45 (6.4)	116.491	<0.001***
**Current self-rating health status**					
Very poor/Poor	11 (1)	30 (5.4)	27 (3.8)	42.087	<0.001
Average	105 (9.9)	72 (12.9)	109 (15.5)		
Good/Very good	948 (89.1)	455 (81.7)	567 (80.7)		
**Chronic illness**					
Yes	223 (21)	127 (22.8)	146 (20.8)	0.937	0.626
**Close contact with an individual with confirmed infection with COVID-19**					
Yes	6 (0.6)	130 (23.3)	109 (15.5)	227.415	<0.001***
**Indirect contact with an individual with confirmed infection with COVID-19**					
Yes	16 (1.5)	127 (22.8)	49 (7)	221.011	<0.001***
**Contact with an individual with suspected infection with COVID-19**					
Yes	76 (7.1)	182 (32.7)	98 (13.9)	185.209	<0.001***
**Contact with infected material**					
Yes	165 (15.5)	107 (19.2)	34 (4.8)	65.560	<0.001***
**Travel to high-risk countries with COVID-19**					
Yes	29 (2.7)	41 (7.4)	11 (1.6)	34.405	<0.001***

* p<0.05, **p<0.01, ***p<0.001.

Table S2. Comparison of knowledge and concerns related to COVID-19 between the first, second and third-survey respondents (n =2324).

Knowledge and concerns related to COVID-19	The first-survey respondents (N = 1064)	The second-survey respondents (N = 557)	The third- survey respondents (N = 703)	Chi-square statistics (X^2)	p-value
	N (%)	*N (%)*	*N (%)*		
**Route of transmission**					
**Droplets**					
Yes	1055 (99.2)	551 (98.9)	682 (97)	13.797	0.001**
**Transmitted through touch with infected person**					
Yes	565 (53.1)	257 (46.1)	294 (41.8)	22.619	<0.001***
**Contact with contaminated objects**					
Yes	903 (84.9)	420 (75.4)	459 (65.3)	91.386	<0.001***
**Transmitted through food**					
Yes	173 (16.3)	87 (15.6)	121 (17.2)	0.601	0.740
**Do not know**					
Yes	4 (0.4)	9 (1.6)	25 (3.6)	26.621	<0.001***
**Satisfaction with the amount of health information about COVID-19**					
Satisfied	468 (44)	123 (22.1)	247 (35.1)	120.122	<0.001***
Not satisfied	204 (19.2)	221 (39.7)	151 (21.5)		
Do not know	392 (36.8)	213 (38.2)	305 (43.4)		
**How often do you check information regarding the status of the coronavirus pandemic?**					
Several times a day		57 (10.2)	5 (0.7)	183.219	< 0.001***
Once a day	NA	208 (37.3)	113 (16.1)		
Once every few days		192 (34.5)	267 (38)		
Difficult to say		100 (18)	318 (45.2)		
**Do you know what to do if you suspect coronavirus infection?**					
Yes	980 (92.1)	469 (84.2)	672 (95.6)	52.510	<0.001***
No	15 (1.4)	17 (3.1)	7 (1)		
Difficult to say	69 (6.5)	71 (12.7)	24 (3.4)		
**Do you follow the news from other countries regarding the**					

**development and course of the pandemic?**					
Yes	657 (61.7)	152 (27.3)	116 (16.5)	503.507	< 0.001***
No	46 (4.3)	126 (22.6)	241 (34.3)		
Occasionally	361 (33.9)	279 (50.1)	346 (49.2)		
**Concerns about COVID-19 epidemic**					
**Concerns about lack of healthcare in case of infection with COVID-19**					
Yes	627 (58.9)	375 (67.3)	353 (50.2)	37.745	<0.001***
**Concerns about own health status if infected with COVID-19**					
Yes	437 (41.1)	242 (43.4)	333 (47.4)	6.831	0.033*
**Concerns about health status of family members if infected with COVID-19**					
Yes	899 (84.5)	430 (77.2)	513 (73)	36.064	<0.001***
**Concerns about likelihood of surviving if infected with COVID-19**					
Yes	345 (32.4)	136 (24.4)	215 (30.6)	11.370	0.003**
**Concerns about economic impacts of coronavirus**					
Yes	530 (49.8)	262 (47)	273 (38.8)	20.984	<0.001***
**Concerns about unemployment**					
Yes	233 (21.9)	108 (19.4)	112 (15.9)	9.609	0.008**
**Concerns about incorrect diagnosis of COVID-19**					
Yes	729 (68.5)	121 (21.7)	150 (21.3)	520	<0.001***
**Concerns about extended epidemic of COVID-19**					
Yes	666 (62.6)	247 (44.3)	324 (46.1)	69.541	<0.001***
**Concerns about re-lockdown**					
Yes	NA	NA	332 (47.2)	NA	NA
**No concerns**					
Yes	30 (2.8)	18 (3.2)	44 (6.3)	14.189	<0.001***

* $p<0.05$, **$p<0.01$, ***$p<0.001$.

Table S3. Comparison of precautionary measures related to COVID-19 between the first, second and third-survey respondents (n =2324).

Precautionary measures	The first-survey respondents (N = 1064)	The second-survey respondents (N = 557)	The third- survey respondents (N = 703)	Chi-square statistics (X^2)	p-value
	N (%)	*N (%)*	*N (%)*		
**Precautionary measures**					
**Wearing mask and protective gloves**					
Yes	303 (28.5)	506 (90.8)	599 (85.2)	851.366	<0.001***
**Covering mouth when coughing and sneezing**					
Yes	791 (74.3)	418 (75)	573 (81.5)	13.247	0.0014**
**Washing hand with soap and water**					
Yes	1016 (95.5)	484 (86.9)	618 (87.9)	46.423	<0.001***
**Using disinfectants**					
Yes	721 (67.8)	411 (73.8)	452 (64.3)	13.043	0.0015**
**Self-isolating at home**					
Yes	833 (78.3)	198 (35.5)	199 (28.3)	513.267	<0.001***
**Eating healthy**					
Yes	547 (51.4)	252 (45.2)	342 (48.6)	5.645	0.059
**Social distancing**					
Yes	817 (76.8)	278 (49.9)	196 (27.9)	419.470	<0.001***
**Not applicable**					
Yes	16 (1.5)	16 (2.9)	23 (3.3)	6.538	0.038*
**Wearing a mask regardless of the presence or absence of symptoms**					
Yes	371 (34.9)	519 (93.2)	618 (87.9)	780.035	<0.001***
**Are you convinced of the need to wear a mask?**					
Yes	NA	393 (70.6)	466 (66.3)	2.611	0.106
**How much are you convinced about the effectiveness of the mask as a protective measure**					
Fully convinced		159 (28.5)	141 (20.1)	14.242	0.007**
Fairly convinced		152 (27.3)	220 (31.3)		
Somewhat convinced	NA	126 (22.6)	160 (22.8)		
Fairly unconvinced		56 (10.1)	94 (13.4)		
Completely unconvinced		64 (11.5)	88 (12.5)		
**Average number of hours staying at home per day to avoid COVD-19**					
20-24 hours	695 (65.3)	220 (39.5)	192 (27.3)	278.314	<0.001***
10-19 hours	279 (26.2)	223 (40)	324 (46.1)		
0-9 hours	90 (8.5)	114 (20.5)	187 (26.6)		
**I am vaccinated against COVID-19**					
Yes	NA	NA	559 (79.5)	NA	NA

* $p<0.05$, **$p<0.01$, ***$p<0.001$.
